# Event and Comment

**Published:** 1905-08-15

**Authors:** 


### DENTAL REGISTER.

Vol. LIX. August 15, 1905. No. 8.

### EVENT AND COMMENT.

### Can Dental Operations be Made More Attractive to Patients?

If there is anythig that people generally dislike to do it is to visit the dentist. Many people purposely let their teeth go to ruin rather than submit to the necessary operations required for saving them. Is is only the pride of better looks and the fact that mastication may be more thoroughly performed, that keep some patients coming to the dentist. They all dread it worse than having a photograph made, or taking a dose of quinine. A ray of hope has recently come into the gloom which hovers over every dentist in consequence of the uncomplimentary remarks continually made about his services by his patients, and from a source which promises to rob dentistry of some of its worst terrors for the patient. The high degree of success now to be secured from dental anesthetic in rendering some operations painless that were almost brutal in the intense suffering incident to their performance is one cause for the new hope. Another is to be looked for in the direction of a decreasing need of the kind of operations formerly so painful. Because of the effort now being made to prevent decay of the teeth and disease of the gums by prophylactic measures. The patients of Dr. Smith are said to look forward with great pleasure to the day when they are to visit him and have their teeth polished and their gums massaged.

In the paper by Dr. Wright, published in this issue, which every one ought to read, he makes the prediction that the future dentist can and will give much time and attention to the scientific massaging of the teeth and gums of children during the periods of jaw and tooth growth, with the intention of securing normally developed jaws and the teeth all erupted into their proper places.

There are many indications that the dentistry of the future may not be altogether reparative, but that prophylaxis shall be the most important feature of the dentists practice. There can be no question but that prophylaxis as advocated by Dr. Smith, will prevent decay of the teeth and also prevent the loss of teeth from pyorrhea alveolaris. If will also cure the majority of cases of this troublesome disease that have caused many a dentist to feel that it was a useless undertaking to treat some cases of this trouble. There are practitioners now claiming to cure nine tenths of the cases that come to them by this means only, without using medicines of any sort. If, as Dr. Wright suggests, we can bring a perfectly developed set of teeth into perfect jaws, and they can be kept so, it would look as though orthodontia would become another of the lost arts. It seems to us as though we were really on the eve of a revolution in the practice of our profession, and that with the improved filling materials, the porcelain inlay and the expert orthodontists we shall soon seek to provide our patients with perfectly occluded teeth that shall be kept clean, and the mouth, instead of a breeding ground for inumerable pathogenic micro-organisms, shall perform its function of food trituration in a perfect manner and as a consequence perfect digestion and good health shall prevail, and a visit to the dentist shall have no terrors for even the most timid.

**Former Ideals Contrasted.**

The idea of filling teeth in years gone was to stop decay of the teeth, and no special effort was made to restore contour. The old soft foil fillers made good stoppers and prevented decay but they did not provide good grinders.

Later methods have sought to correct this fault. For years we have stopped the teeth and after a fashion restored contours. We have now reached the stage where we add the use of such materials as shall prevent decay, restore contours and also produce smooth surfaces, which can be kept free from extraneous deposits. The porcelain inlay joins hands with prophylaxis. If it can be made durable and restore the cusps and angles as well as gold, it has a great field of usefulness and will rank high as the ideal filling material.

**Recognition of the Home-trained Dentist.**

Dr. A. H. Peck in discussing an address on dental education before the Chicago Dental Society, makes the following declaration:

"I am as steadfast a believer in college training as any one present. I would, were it possible, have every individual receive a college training, the better to fit him for his vocation, no matter, in the main, what it might be. At the same time, I also firmly believe if one has the requisite ability to successfully engage in his chosen vocation, no matter whether gained is a college or out of a college, to him the right to use that ability is an inherent one, and from him no human power has the right to take it."

Viewed from the standpoint of sentiment on charity toward an individual, who, because of his circumstances has been unable to prepare himself for practice by the methods almost universally conceded to be the best, namely a college course, this statement can easily be endorsed by every one. But when we consider the possibilities of admitting to a profession where the most vital interests are to be considered, and where an improperly or partially trained practitioner can do so much injury to innocent people, it becomes more than a question of sentiment. It becomes our duty to enact legislation that shall make it difficult, if not impracticable, for an indifferently trained person to become a legalized practitioner. Some one will say that colleges do not always graduate fully trained men, and that many a self-made dentist is superior to the average college graduate. In answer we think we can safely say that there is logically more reason to believe that the college graduate will successfully practice his profession than that the self-trained man will do so. Take two men of nearly equal character and capacity, and the college man will be far ahead of the self-educated man after a period of ten years. The college is organized to train men for this purpose and every year it is becoming better equipped for its work. It is establishing a standard of attainment and should be encouraged by the legislative departments in order that it may continually strive to better its output.

### Shall we Graduate on the Time, or the Educational Basis?

In discussing the address of President James, Dr. Cigrand says: "The idea that colleges shall graduate students as an army at a certain specified time, called commencement day at the end of a three-years course of study, is a serious mistake in our educational system. The student should be graduated just as soon as the faculty is convinced that he knows his curriculum, and no sooner. It is a mistake to have any subject taught in a curriculum that is not essential, giving students the option of slighting one subject that he may develope a higher attainment in some other. No student should be graduated who has not mastered every subject."

This is high ground and a very proper position to take in this matter, and now that the National Board of Examiners has set a minimum time for the colleges, no student can be graduated in less time. Should the colleges take the advance step suggested by Dr. Cigrand and compel students to remain in college until they have mastered every subject of the curriculum, they would certainly have no end of trouble with their students, but their output of graduates would have little trouble in passing the state board examinations. This would be real progress in dental education, and would very soon place the colleges in more confidential relations with the profession.

